# Quadriceps muscle activity during commonly used strength training exercises shortly after total knee arthroplasty: implications for home-based exercise-selection

**DOI:** 10.1186/s40634-019-0193-5

**Published:** 2019-07-02

**Authors:** Thomas Linding Jakobsen, Markus Due Jakobsen, Lars Louis Andersen, Henrik Husted, Henrik Kehlet, Thomas Bandholm

**Affiliations:** 1 Physical Medicine & Rehabilitation Research – Copenhagen (PMR-C), Department of Physical and Occupational Therapy, Copenhagen University Hospital, Hvidovre, Denmark; 2 Lundbeck Foundation Centre for Fast-track Hip and Knee Arthroplasty, Copenhagen University Hospital, Hvidovre, Denmark; 3 Department of Orthopedic Surgery, Copenhagen University Hospital, Hvidovre, Denmark; 4 National Research Centre for the Working Environment, Copenhagen, Denmark; 5 Clinical Research Centre, Copenhagen University Hospital, Hvidovre, Denmark; 6 Section for Surgical Pathophysiology 7621, Rigshospitalet, University of Copenhagen, Copenhagen, Denmark; 7 Section for Orthopaedic and Sports Rehabilitation (SOS-R), Health Centre Nørrebro, City of Copenhagen, Copenhagen, Denmark; 8 Sport Sciences, Department of Health Science and Technology, Aalborg University, Copenhagen, Denmark

**Keywords:** Total knee arthroplasty, Strength training, Electromyography, Rehabilitation, Exercise

## Abstract

**Background:**

In the early phase after a total knee arthroplasty (TKA), patients experience multi-level weakness in the operated leg, which is caused primarily by reduced central nervous system (CNS) activation failure of the muscles - especially the knee extensors (quadriceps muscle). Whether similar levels of neuromuscular activity of the muscles in the operated leg, elicited during strength training exercises in machines, can be reached during strength training exercises in more simple forms is unknown. Many clinicians are faced with the problem of not having strength training equipment at their institution or having to prescribe simple strength training exercises for home-based training. Therefore, the purpose of this study was to determine which strength training exercises that activated the muscles in the operated leg the most after TKA. The hypothesis was that strength training exercises performed in machines would elicit higher levels of voluntary peak quadriceps and hamstring muscle activity than strength training exercises performed in more simple forms, using elastic bands or the patients’ own body weight.

**Methods:**

A cross-sectional electromyographic study investigated voluntary peak muscle activity in the operated leg during 6 different strength training exercises. Twenty-four patients, who received a TKA 4 to 8 weeks earlier, performed the exercises in a randomized order, using a pre-determined loading of 10 RM (repetition maximum). Voluntary peak muscle activity (%EMGmax) was calculated for the quadriceps and hamstring muscles for each exercise.

**Results:**

Knee extensions with elastic band showed significantly higher voluntary peak quadriceps muscle activity than knee extensions in machine (93.3 vs. 74.9; mean difference, 18.3 %EMGmax [95% confidence interval (CI), 11.7 to 24.9]; *P* < 0.0001). Similarly, one-legged squat (and sit to stand) elicited higher voluntary peak quadriceps muscle activity than leg press in machine (86.7 vs. 66.8; mean difference, 19.9 %EMGmax [95% CI, 14.8 to 25.0]; *P* < 0.0001).

**Conclusions:**

Strength training exercises in more simple forms elicited higher voluntary peak quadriceps muscle activity than strength training exercises in machines early after TKA. Consequently, simple home-based strength training exercises using e.g. elastic bands or the patients’ own bodyweight should be considered to alleviate muscle strength losses early after TKA.

**Trial registration:**

ClinicalTrials.gov identifier: NCT01708980.

**Electronic supplementary material:**

The online version of this article (10.1186/s40634-019-0193-5) contains supplementary material, which is available to authorized users.

## Background

Total knee arthroplasty (TKA) is performed to alleviate knee pain and disability related to end-stage knee osteoarthritis. The number of TKA operations has increased in the developed countries (Carr et al. 2012) with an estimated 693,400 operations performed in the US in 2010 (Williams et al. 2015). In the early phase after TKA, patients experience considerable weakness of several muscles in the operated leg (Stevens-Lapsley et al. 2010; Holm et al. 2010; Piva et al. 2011; Judd et al. 2012), which is caused primarily by reduced central nervous system (CNS) activation failure of the muscles - especially the knee extensors (Mizner et al. 2005a; Rice and McNair 2010). This pronounced loss of knee-extension strength attributed to the quadriceps muscle may compromise important daily activities such as climbing stairs and rising from a chair (Eriksrud and Bohannon 2003; Mizner et al. 2005b; Holm et al. 2010).

Consequently, exercise-based rehabilitation encompassing strength training exercises is common practice after TKA (Westby et al. 2014; Artz et al. 2015). According to the latest systematic review on the effectiveness of physiotherapy exercise on patient-reported physical function, exercise-based supervised outpatient rehabilitation seems superior to no or minimal rehabilitation (Artz et al. 2015). Recent studies have found home-based rehabilitation to be equally effective as out-patient rehabilitation (Han et al. 2015; Artz et al. 2015), or one-to-one rehabilitation not to be superior to group or home-based rehabilitation after TKA (Ko et al. 2013). However, none of the home-based rehabilitation programs (Ko et al. 2013; Han et al. 2015) included strength training exercises.

Rehabilitation including strength training exercises, implemented shortly following TKA, has proven to be feasible without exacerbating postoperative symptoms e.g. knee joint effusion and knee pain (Jakobsen et al. 2011; Bade and Stevens-Lapsley 2011; Bandholm et al. 2014; Jakobsen et al. 2014b). Machine-based strength training exercises has been promoted to enhance muscle strength and functional performance in patients with TKA (Bade and Stevens-Lapsley 2011). Recently, more simpler forms of strength training using exercises that can be implemented at home have been shown to activate the lower limb muscles, e.g. the quadriceps muscle, at a similar level as more machine-based strength training exercises in healthy subjects (Jakobsen et al. 2012; Aboodarda et al. 2016) and in patients with chronic stroke (Vinstrup et al. 2016, 2017). The question is if this is also the case in patients shortly following a TKA?

Therefore, the purpose of this study was to determine which strength training exercises that activated the muscles in the operated leg the most after TKA. We compared the voluntary peak quadriceps and hamstring muscle activity during strength training exercises performed in machines to that during strength training exercises in more simple forms, using elastic bands or patients’ own body weight. The hypothesis was that strength training exercises performed in machines would elicit higher levels of voluntary peak quadriceps and hamstring muscle activity than strength training exercises performed in more simple forms.

## Methods

### Study design and patients

This was a descriptive, cross-sectional electromyographic (EMG) study that followed the “Strengthening the reporting of observational studies in epidemiology” (STROBE) reporting guidelines (Vandenbroucke et al. 2014), using the checklist for cross-sectional studies. The study compared voluntary peak quadriceps and hamstring muscle activity in the operated leg during strength training using machines and during strength training in more simple forms, using elastic bands or patients’ own body weight, 4 to 8 weeks after unilateral primary TKA.

Twenty-four patients were recruited by consecutive sampling from 4 different out-patient rehabilitation centers (Hvidovre, Brøndby, Vanløse, and Vesterbro), having been operated at one of 4 different hospitals (Bispebjerg, Amager/Hvidovre, Gentofte and Frederiksberg) in the Copenhagen Area, from August 2012 to February 2013. Collection of outcomes was performed at the Department of Physical and Occupational Therapy, Copenhagen University Hospital, Hvidovre, Denmark.

The inclusion criteria were: 18–80 years of age, operated 4 to 8 weeks earlier with a unilateral primary TKA, informed consent, and able to understand and speak Danish. The exclusion criteria were: neuromusculoskeletal disorder(s) that required a special physical rehabilitation program, alcohol or medication abuse, and unwillingness to participate.

### Test procedure

Outcomes were assessed twice; an initial familiarization session at which the weight loads/patients’ body position during the strength training exercises were determined, followed by an experimental session 72 h later at which all primary outcomes were collected by the 2 investigators. Secondary outcomes were assessed before, during, and after both the familiarization and the experimental sessions. Prior to the familiarization session, baseline characteristics were assessed including knee joint ROM, and knee joint effusion assessed by measurement of the knee joint circumference (Jakobsen et al. 2010). Muscle soreness in the operated leg was registered (verbal reported) right before the experimental session to indicate any persistent soreness from the familiarization session.

#### Familiarization session

During the familiarization session, patients went through the exercise protocol which consisted of careful instruction in the 6 different strength training exercises including determination of the load in kilograms corresponding to 10 repetition maximum (RM) for each exercise. The determination of 10 RM was usually achieved within 2 to 3 attempts to the best estimate of the investigator. The 6 exercises were: knee extensions in machine (KEM), knee extensions with elastic band (KEE), leg press in machine (LEP), sit-to-stand (STS), one-legged squat (OSQUAT) and straight leg raise (SLR) (Additional file 1). All exercises have previously been used in exercise programs for patients with knee OA or TKA (Moffet et al. 2004; Ageberg et al. 2010; Bade et al. 2010; Jakobsen et al. 2011; Bade and Stevens-Lapsley 2011).

The selection of strength training exercises focused on the voluntary activity of the quadriceps muscle to restore reduced knee-extension strength (Mizner et al. 2005a; Holm et al. 2010) as it is related to reduced functional performance early after TKA (Mizner et al. 2005a; Holm et al. 2010). The primary rationale was to compare similar strength training exercises performed in machines or in more simple forms using elastic bands or patients’ own body weight. For the open chain strength training exercise (knee-extensions), the machine-based exercise (KEM) was considered the gold-standard and compared to a simple exercise using elastic band(s) (KEE). The focus was specifically to activate the quadriceps muscle voluntarily (Andersen et al. 2006). Similarly, for the closed chain strength training exercise (leg press), a machine-based exercise (LEP) was considered the gold standard and compared to 2 simple exercises (STS and OSQUAT), using the patients’ own body weight. For the closed chain strength training exercises, the focus was to activate, in addition to the quadriceps muscle as well as several other lower leg muscles with reported strength deficits following TKA (Judd et al. 2012).

The order of exercises was randomized and performed unilaterally using the correct strength training technique. All exercises were carefully standardized, and exercise descriptors were noted, such as weight loads and the patients’ positions and foot wear during exercises. Every repetition started from full active knee extension (minimum 10 degrees of knee flexion) to a minimum of 80 degrees of knee flexion and returned to full active knee extension. All exercises followed this repetition cycle, except the straight leg raise exercise. Patients had no rest between repetitions. To control for time under tension, patients followed a pre-recorded audio file with modes of 2, 3, 2, 3 s of isometric, eccentric, isometric and concentric contractions, respectively. During the straight leg raise, patients flexed the hip until the leg was aligned with a 50-cm long piece of tape on the wall adjacent to the examination couch, indicating that the patient had flexed 60 degrees in the hip. The amount of knee joint ROM was based on visual estimation, and in some cases assessed using a large (moveable arms of 30 cm) universal plastic goniometer (Jakobsen et al. 2010). Patients were instructed to align hip, knee cap and 2nd and 3rd toe in a straight line during the exercises. If OSQUAT and STS could not be performed solely on the operated leg, the patients were allowed to partially weight-bear with the non-operated leg. To secure standardization, the patients’ load on the non-operated leg was registered on a weight scale fitted into a custom-made wooden platform, on which the OSQUAT and STS were performed. Additionally, the toe-to-toe distance between legs was noted.

#### Experimental session

At least 72 h after the familiarization session, the experimental session was carried out to avoid delayed onset muscle soreness. The EMG setup was prepared, before the patients performed the maximal voluntary isometric contractions (MVICs) and the strength training exercises.

The recommended electrode placement for the use of surface EMG over medial (VM) and lateral (VL) vastus of the quadriceps muscle, biceps femoris (BF) muscle, semitendinosus (ST) muscle and reference point over the patella was found and confirmed by both investigators (Perotto and Delagi 2005). The skin was shaved at the designated electrode locations and prepared with fine 240-grit sand paper and cleaned with ethanol. The surface electrodes (DE-2.1, Delsys, Boston, MA, USA) with 2 mm × 1-cm long parallel bars, and 1 cm distance between bars, were prepared with electrode gel. The EMG electrode interface was made from medical grade adhesive. The knee joint ROM during the exercise was measured using a large electronic goniometer (Goniometer Biosignal Sensors Delsys, Boston, MA, USA). The goniometer was positioned at the lateral aspect of the leg with the fulcrum placed over the lateral epicondyle of the femur, and the ends of the goniometer pointing towards the greater trochanter and the lateral malleolus (Jakobsen et al. 2010). The EMG electrodes, the electronic goniometer and connecting electrode wires were fixated to the skin with medical tape to maximize EMG and goniometer signal quality during contractions. The pre-amplified (built into the electrode) EMG signal was sent to the main amplifier via an input module, where it was band-pass filtered (15–450 Hz) using a common-mode rejection ratio of 92 dB. The signals were sampled at 1 kHz with at 16-bit A/D converter (6036E, National Instruments, Austin, TX, USA) and transferred to a personal computer, where the raw signal quality was visual assessed and approved for further analysis (EMGworks 3.7 Acquisition, Delsys, Boston, MA, USA).

Patients warmed up 3 min on a stepping machine at a self-selected intensity followed by 3 unilateral MVICs in knee extension and flexion at a knee joint angle of 60 degrees. Strong verbal encouragement was provided during contractions, which were separated by 3-min pauses. The order of knee flexion and extension was randomized. After the 3 MVICs per movement direction, the patients performed 4 repetitions of each of the strength training exercises with the pre-determined 10 RM loading to avoid fatigue during the set. The exercise order was the same as randomized at the familiarization session. Three-minute rests separated the exercises to prevent muscular fatigue. If patients did not perform the exercise correctly, or stopped the exercise, the patients were asked to repeat 4 repetitions of the exercise after a short break. This happened once during the OSQUAT.

### Data collection and analysis

#### Primary outcome

During off-line analysis, all raw EMG signals obtained during MVICs as well as during the exercises were digitally high pass filtered using a Butterworth 4th order high-pass filter (10 Hz cutoff frequency) and smoothed with a symmetrical root mean square filter (500 ms constant) (MatLab, The Mathworks Inc., Natick, MA, USA). Voluntary peak muscle activity of the filtered EMG signal was determined for each repetition of each muscle, and subsequently normalized to the filtered EMG obtained during MVIC (Jakobsen et al. 2014a). Accordingly, the normalized EMG values were expressed as a percentage of the EMGmax of each respective muscle (%EMGmax). Each of the 4 repetitions during the strength training exercises (except for SLR) was identified using data from the electronic goniometer. The first repetition of each exercise was omitted from the analysis due to a pre-determined risk of poor EMG quality. The goniometer signal was digitally lowpass filtered using a 4th order zero-lag Butterworth filter (3 Hz cutoff frequency) (Jakobsen et al. 2012). Voluntary peak muscle activity during the SLR was determined as the highest filtered EMG signal of all 4 repetitions.

#### Secondary outcomes

Knee pain was measured with a 0–100 mm visual analog scale (VAS) with end points of “no pain” and “worst pain imaginable” (Breivik et al. 2008). Before and after both the familiarization and experimental session, knee pain at rest was assessed while patients were seated with the operated knee in an approximately 60° of knee flexion. Knee pain and perceived exertion, using the Borg scale ranging from 6 (no exertion at all) to 20 (maximal exertion) (Borg 1970), during the strength training exercises were measured by the patients’ recall immediately after the exercise.

### Statistical analyses

The sample size estimation was calculated to detect a difference for the primary outcome, normalized voluntary peak EMG (%EMGmax) activity of the quadriceps muscle (pooled data from vastus lateralis (VL) and vastus medialis (VM)), and between knee-extensions performed in machine and with elastic bands, respectively. Based on previous work in healthy subjects (standard deviation of pooled VL and VM %EMGmax activity data of 72% (Andersen et al. 2006) and 32% (Jakobsen et al. 2012)) and pilot data acquired from patients with TKA (*n* = 3, standard deviation of pooled VL and VM %EMGmax activity data of 20% (data are not shown)), a sample of 20 patients was required for a paired t-test (2-tailed analysis) with a significance level of 5% and a power of at least 80% to detect a minimal relevant difference of 10% %EMGmax activity between exercises, and assuming a mean common standard deviation of 43 %EMGmax and an intraclass correlation coefficient of 0.94 (Andersen et al. 2008). The minimal relevant difference in voluntary muscle activity (10 %EMGmax) was determined from the strength training literature, where recommendations are often given in increments of 10% points (Kraemer et al. 2002). To account for dropouts, 24 patients were included.

The primary statistical analysis was a linear mixed model analyzing the difference in voluntary peak quadriceps muscle activity during strength training exercises performed in machines and using elastic bands/body weight, respectively. The knee extensions in machine (KEM) was compared with knee extensions with elastic band (KEE), and leg press in machine (LEP) was compared with sit-to-stand (STS) and one-legged squat (OSQUAT). The model was fitted in the Statistical Analysis System (SAS) version 9.4 (SAS Institute Inc., Cary, NC, USA) using the “PROC MIXED” procedure with patients as a random factor and exercise (KEM, KEE, LEP, STS, OSQUAT), muscle (VM, VL, ST, BF) and number of repetitions (1 to 3) as fixed factors. Voluntary peak quadriceps muscle activity (VM and VL data pooled) was the dependent variable and differences between exercises are presented as least square mean values with corresponding 95% confidence intervals (CI). The PROC MIXED procedure estimates values of potentially missing data. Using the same statistical approach as described for the primary statistical analysis, a secondary statistical analysis investigated the differences between the voluntary peak quadriceps muscle activity during closed isotonic chain strength training exercises (LEP, STS, OSQUAT), open isotonic chain strength exercises (KEM, KEE) and open isometric strength(ening) exercise (SLR), respectively. Furthermore, the LEP was compared with both STS and OSQUAT, where the dependent variable was voluntary peak hamstring muscle activity (semitendinosus (ST) and biceps femoris (BF) data pooled). The pooled data used in the analysis derived from the normalized voluntary peak EMG (%EMGmax) value from each repetition (3) for each muscle (VM, VL, ST and BF). The differences are presented as least square mean values with corresponding 95% CI.

One-way repeated ANOVA with Tukey-Kramer adjustment was used to assess difference in the perceived exertion during exercise, and for the non–normally distributed knee pain data the Kruskal-Wallis test was used.

All continuous data, baseline characteristics and outcome measures were assessed for normal distribution using Shapiro-Wilk test, and visual examination of probability plots and histograms. Additionally, visual interpretation of the residuals for all voluntary peak muscle activity data was done during the PROC MIXED procedure. Means with standards deviations (±1SD) or standard error of the mean (±SE), and medians with interquartile ranges (IQR) were expressed for normally distributed and non-normally distributed data, respectively. Categorical data were presented as counts with percentages. All statistical analyses were conducted using SAS. Sample size estimation was performed using the SAS Power and Sample Size application (PSS). All baseline data, knee pain and perceived exertion data were double entered and validated in EpiData Entry, version 3.1 (Epidata, Odense, Denmark). A *p*-value of 0.05 was considered significant.

## Results

### Patients

**Fig. 1:**
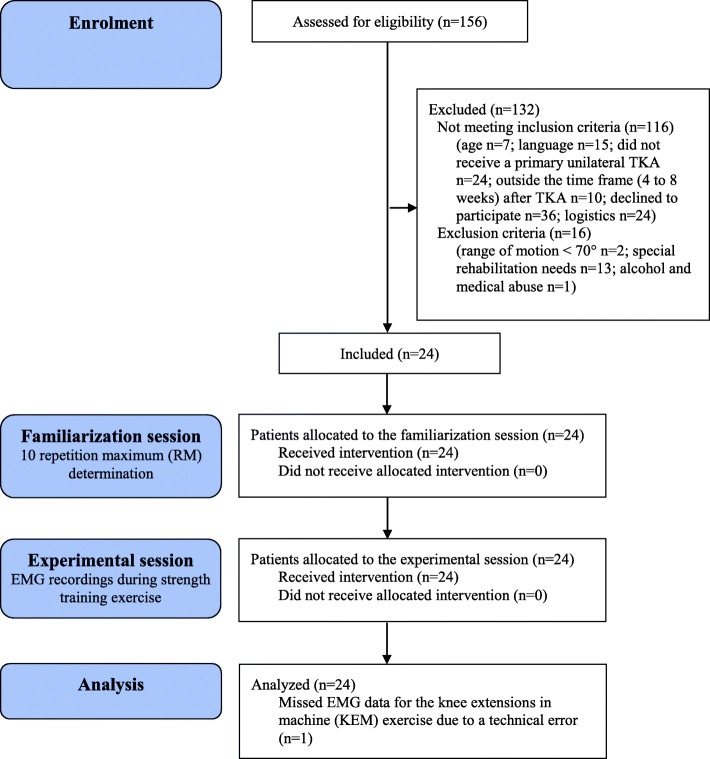
Patient flow diagram

One-hundred-and-fifty-six patients with TKA were assessed for eligibility (Fig. 1). In total, 24 patients were enrolled in the study. One set of EMG data from a patient performing knee extensions in machine (KEM) was excluded due to technical error (*n* = 1). This was decided during visual inspection of the EMG data prior to data analysis. Baseline characteristics of the final sample are shown in Table 1. The TKA prostheses were the cemented AGC (Zimmer Biomet, Warsaw, IN, USA) (*n* = 11), uncemented PFC Sigma (Johnsson & Johnsson, Warsaw, IN, USA) (*n* = 4), cemented Vanguard CR (Zimmer Biomet, Warsaw, IN, USA) (*n* = 2), cemented NexGen (Zimmer Biomet, Warsaw, IN, USA) (*n* = 3), and uncemented NexGen CR-Flex (Zimmer Biomet, Warsaw, IN, USA) (*n* = 4). Patients experienced no or mild knee pain (Treede et al. 2019) at rest before (median VAS-mm = 0 (IQR 0–1)) and after (median VAS-mm = 0 (IQR 0–0)) the familiarization session, and before (median VAS-mm = 0 (IQR 0–2)) and after (median VAS-mm = 0 (IQR 0–0)) the experimental session. Muscle soreness was experienced by 17 patients (71%) after the familiarization session and was located to the quadriceps muscle (*n* = 9), hamstrings (*n* = 3), gluteal muscles (*n* = 9), hip flexors (*n* = 4) and calf muscles (*n* = 8).

**Table 1:** Baseline characteristics (*n* = 24)

Age, years, mean (SD)	67 (8)
Men, no (%)	10 (42)
Body weight, kg, mean (SD)	85.7 (14.3)
Height in cm, mean (SD)	172.3 (9.5)
Body mass index, mean (SD)	28.8 (3.7)
Other diseases, no (%)	22 (92)
Operated knee (right/left), no (%)	10 (42)/ 14 (58)
Opposite knee operated, no (%)	12 (50)
Fall(s) within the last ½ year, no (%)	5 (21)
Pain medication before testing, no (%)	23 (96)
No. of rehabilitation sessions before testing, mean (SD)	5.8 (2.4)
Strength training part of the rehabilitation, no (%)	24 (100)
Rehabilitation center (Van/VKV/Hvh/Br), no (%)	11 (46)/ 5 (21)/ 6 (25)/ 2 (8)
Days after operation, mean (SD)	38.3 (9.4)
Knee joint circumference, cm, mean (SD)	45.5 (2.9)
Knee PROM extension, degrees, mean (SD)^a^	2.5 (6.2)
Knee PROM flexion, degrees, mean (SD)	112.3 (15.5)

*No* Number, *SD* 1·Standard deviation, *IQR* Interquartile range, *Van* Vanløse, *VKV* Vesterbro/Kgs. Enghave/Valby, *Hvh* Hvidovre and *Br* Brøndby, *PROM* passive range of motion (ROM)

^a^ Positive knee extension scores indicate ROM limitation (inability to reach the 0° starting position)

### Outcome measures

**Fig. 2:**
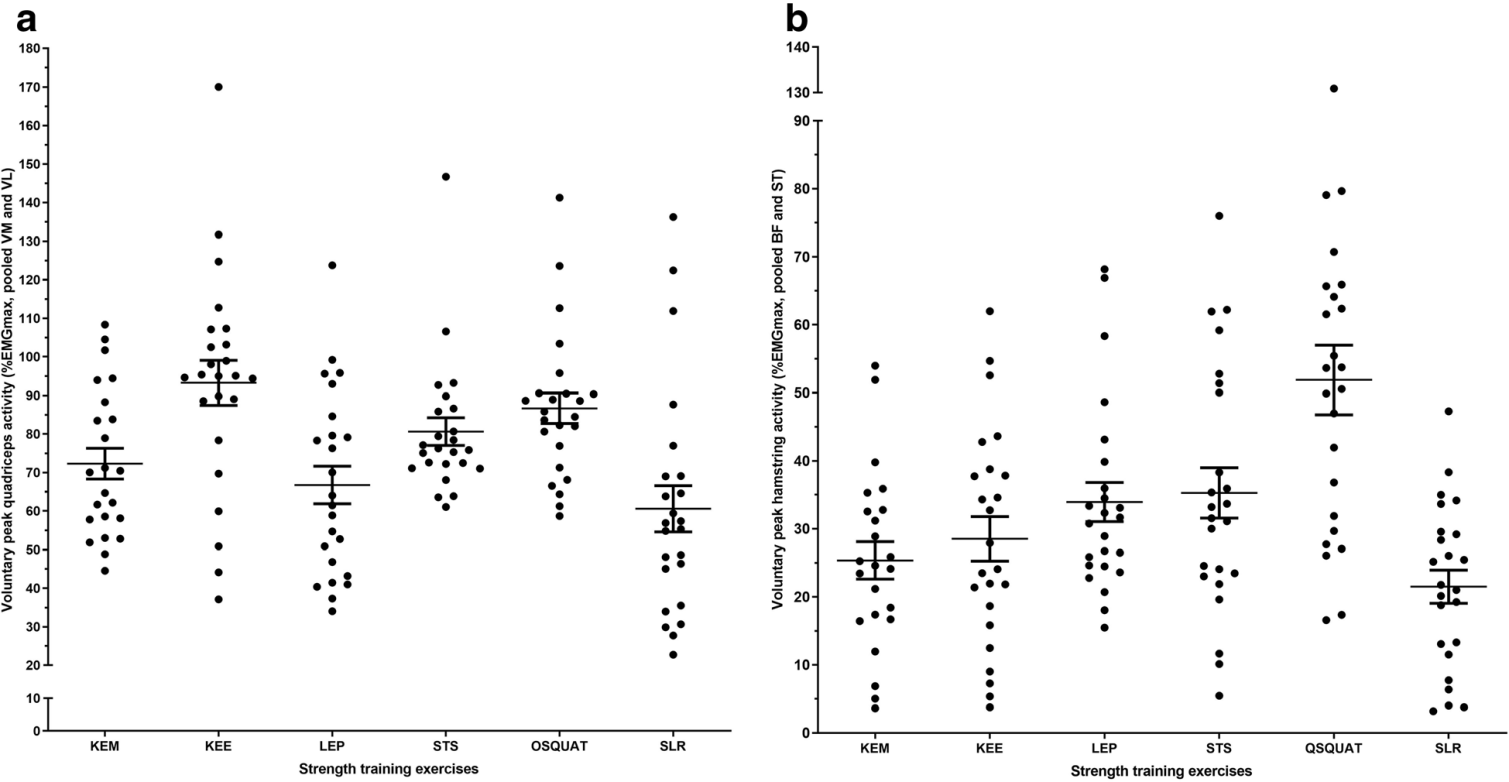
Voluntary peak (**a**) quadriceps and (**b**) hamstring muscle activity during the 6 strength training exercises. Scatterplot with means ±1·SE (standard error of the mean) bars. KEM = Knee extensions in machine; KEE = Knee extensions with elastic band; LEP = Leg press in machine; STS=Sit-to-stand; OSQUAT = One-legged squat; SLR = Straight leg raise

Voluntary peak muscle activity for VM, VL, BF, ST, quadriceps (pooled VM and VL data) and hamstrings (pooled BF and ST data), absolute load and toe-to-toe distance between legs, perceived exertion (Borg Scale) and knee pain (VAS-mm) during the 6 strength training exercises at the experimental session are presented in Table 2. Voluntary peak muscle activity for the quadriceps and hamstring muscles during the strength training exercises are shown in Fig. 2.

**Table 2:** Normalized voluntary peak EMG (%EMGmax) activity, absolute load, perceived exertion and knee pain during the strength training exercises

Exercise	VM (%), mean ± SD	VL (%), mean ± SD	Quadriceps (%), mean ± SD	ST (%), mean ± SD	BF (%), mean ± SD	Hamstring (%), mean ± SD	Load on operated leg (kg)^a^/load on non-operated leg (kg)^b^/toe-to-toe distance (cm)^c^/elastic resistance (no., color)^e^, mean ± SD	Perceived exertion (Borg scale, points), experimental session, mean ± SD	Knee pain during exercise (VAS-mm), experimental session, median (IQR)
Knee extensions in machine (KEM)	72.8 (20.1)^d^	71.8 (20.7)^d^	72.3 (19.2)^d^	15.2 (8.6)^d^	35.6 (20.7)^d^	25.4 (13.1)^d^	8.8 (4.0)^a^	12.4 (2.3)	0 (0–26)
Knee extensions with elastic band (KEE)	97.6 (32.4)	88.9 (27.6)	93.3 (28.5)	18.6 (11.0)	38.5 (25.8)	28.5 (16.0)	1 green (n = 3)^e^ 1 blue (*n* = 5)^e^ 2 green (n = 6)^e^ 1 green+ 1 blue (n = 2)^e^ 2 blue (*n* = 6)^e^ 2 green+ 1 red (*n* = 1)^e^ 2 blue (n = 1)^e^	12.3 (2.6)	0 (0–10)
Leg press in machine (LEP)	66.3 (27.3)	67.2 (23.4)	66.8 (23.9)	25.8 (14.5)	42.1 (18.4)	33.9 (14.1)	28.5 (10.8)^a^	12.6 (2.4)	0 (0–18)
Sit-to-stand (STS)	84.9 (26.0)	76.4 (20.4)	80.7 (17.6)	24.8 (12.6)	45.7 (27.1)	35.3 (18.1)	31.8 (11.1)^b^ 17.0 (8.5)^c^	12.6 (2.2)	1 (0–17)
One-legged squat (OSQUAT)	90.44 (26.3)	82.92 (21.8)	86.7 (19.3)	37.6 (13.7)	66.2 (42.2)	51.9 (25.0)	26.9 (12.6)^b^ 71.3 (18.7)^c^	13.3 (2.8)	0 (0–19)
Straight leg raise (SLR)	60.17 (35.3)	61.00 (26.9)	60.6 (29.4)	16.3 (16.0)	26.7 (16.3)	21.5 (12.0)	1.3 (1.2)^a^	12.0 (2.5)	0 (0–8)

^a^Load lifted in kg. ^b^Patients’ own body weight on the non-operated leg measured in kg. ^c^Toe-to-toe distance between front and rear leg in cm. ^d^One missed recording due to a technical problem (*n* = 23), ^e^Colors of the elastic bands with increased resistance from red, green, blue to black. 1 = single elastic band, 2 = doubled elastic band

#### Primary analysis

##### Voluntary peak quadriceps muscle activity during strength training exercises performed in machines and in more simple forms

The primary analysis showed that knee extensions with elastic band (KEE) elicited significant higher voluntary peak quadriceps muscle activity than knee extensions in machine (KEM) (93.3 vs. 74.9; mean difference, 18.3 %EMG [95% confidence interval (CI), 11.7 to 24.9]; *P* < 0.0001). Similar findings were found for the closed chain strength training exercises. The one-legged squat (OSQUAT) demonstrated significantly higher voluntary peak quadriceps muscle activity than leg press in machine (LEP) (86.7 vs. 66.8; mean difference, 19.9 %EMG [95% CI, 14.8 to 25.0]; *P* < 0.0001) and sit-to-stand (STS) (86.7 vs. 80.7; mean difference, 6.0 %EMG [95% CI: 0.9 to 11.1]; *P* = 0.02).

#### Secondary analysis

##### Voluntary peak quadriceps muscle activity during open and closed chain isotonic/isometric strength training exercises

There was no significant difference in voluntary peak quadriceps muscle activity between patients performing an open (pooled data for KEM and KEE) or a closed chain isotonic strength training exercise (pooled data for LEP, STS and OSQUAT) (84.8 vs. 78.0; mean difference, 6.8 %EMG [95% CI, − 1.5 to 15.1]; *P* < 0.11). However, both the open (KEM and KEE) and closed (LEP, STS, OSQUAT) isotonic strength training exercises showed higher voluntary peak quadriceps activity than the open isometric strengthening exercise (SLR) ((open vs. isometric, 84.8 vs. 60.6; mean difference, 24.2 %EMG [95% CI, 15.9 to 32.5]; *P* < 0.0001) and (closed vs. isometric, 78.0 vs. 60.6; mean difference, 17.4 %EMG [95% CI, 9.1 to 25.8]; *P* < 0.0001)).

##### Voluntary peak hamstring muscle activity during closed chain isotonic strength training exercises

The one-legged squat (OSQUAT) elicited significantly higher voluntary peak hamstring (pooled data for BF and ST) muscle activity than leg press in machine (LEP) (51.9 vs. 33.9; mean difference, 18.0 %EMG [95% CI, 13.3 to 22.6]; *P* < 0.0001) and sit-to-stand (STS) (51.9 vs. 35.3; mean difference, 16.6 %EMG [95% CI, 12.0 to 21.3]; *P* < 0.0001).

##### Knee pain and perceived exertion during the strength training exercises

There was no significant difference in knee pain during exercise (*P* = 0.88) or perceived exertion (*P* = 0.57) between the 6 strength training exercises. Generally, patients experienced no (median, 0; interquartile range, 0 to 8 VAS-mm) to mild (median, 1; interquartile range, 0 to 17 VAS-mm) knee pain during the 6 strength training exercises at the experimental session.

### Adverse events

As expected, most patients (71%) experienced delayed onset muscle soreness in the largest muscle groups in the operated leg after the familiarization session. The patients were familiar with this type of muscle pain, as strength (ening) exercises were part of their post-TKA rehabilitation program. There was no significant (*P* = 0.09) difference in knee pain at rest before (median = 0, IQR: 0–15 VAS-mm) and after ((median = 0, IQR: 0–0 VAS-mm) the 6 strength training exercises at the experimental session. Finally, none of the patients withdrew from the study due to discomfort or adverse events.

## Discussion

This cross-sectional, electromyographic study investigated the difference in voluntary peak muscle activity in the operated leg when recorded during 6 different strength training exercises performed early after TKA. The main findings were that 1) simple strength training exercises (knee extensions with elastic band (KEE), sit-to-stand (STS) and one-legged squat (QSQUAT)) elicited higher voluntary peak quadriceps muscle activity than machine-based strength training exercises (knee extensions in machine (KEM) and leg press in machine (LEP)), 2) no differences existed in voluntary peak quadriceps muscle activity between open (KEM, KEE) and closed chain (LEP, STS, OSQUAT) isotonic strength training exercises, 3) isotonic (KEE, KEM, LEP, STS, OSQUAT) showed higher voluntary peak quadriceps muscle activity than isometric (straight leg raise (SLR)) strength training exercise(s), and 4) the OSQUAT had higher voluntary peak hamstring muscle activity compared to the other closed isotonic strength training exercises (LEP, STS).

### Explanation of results

#### Voluntary peak quadriceps muscle activity during machine-based and simple strength training exercises

Contrary to the study hypothesis, we found that simple strength training exercises using elastic bands or patients’ own body weight elicited higher voluntary peak quadriceps activity than machine-based strength training exercises early after TKA. Regarding the higher voluntary peak quadriceps muscle activity in KEE compared to KEM, this observation may be explained by the increase in elastic force generation during the elongation of the elastic band(s) from flexion to the end of knee extension (Aboodarda et al. 2016). The voluntary peak EMG quadriceps muscle activity in the KEM was probably found in lower knee flexion angles (Jakobsen et al. 2012), which may have produced lower absolute EMG levels of voluntary peak quadriceps muscle activity compared to the KEE (Andersen et al. 2006). A recent systematic review with meta-analysis showed no difference in voluntary EMG muscle activity between strength training exercises using elastic resistance devices, such as elastic bands, compared to machines or free weights in an active healthy population (Aboodarda et al. 2016). However, it should be noted that patients with chronic stroke had slightly higher voluntary peak quadriceps muscle activity when performing knee extensions with a load of 10 RM in a machine (KEM) compared to with elastic bands (KEE), which may be due to higher coordination requirement during the KEE (Vinstrup et al. 2016).

The simpler closed chain isotonic exercises STS and OSQUAT provided higher voluntary peak quadriceps muscle activity compared to LEP. Both in EMG evaluation studies (Escamilla et al. 1998, 2001) and in a 10-week randomized study (Rossi et al. 2018), the squat exercise with free weight has shown to be more effective to increase voluntary quadriceps muscle activity and functional performance compared to leg press in machine in healthy populations. On the contrary, in an EMG evaluation study investigating patients with chronic stroke, the chair rise with own bodyweight was inferior in voluntary activating the peak quadriceps muscle compared to uni- and bilateral leg press in machine (Vinstrup et al. 2017). Several reasons may explain the higher voluntary peak quadriceps muscle activity in STS and OSQUAT compared to LEP. First, great care was taken to ensure the pre-determined 10 RM load on the operated leg during the STS and OSQUAT by registering the distance between patients’ feet as well as the absolute load on the non-operated leg. Second, STS and OSQUAT may require more voluntary peak quadriceps muscle activity to stabilize the knee joint during these exercises compared to leg press (Schwanbeck et al. 2009).

#### Voluntary peak quadriceps muscle activity during open and closed chain isotonic/isometric strength training exercises

The open (KEE, KEM) chain isotonic strength training exercises did not elicit higher voluntary peak quadriceps muscle activity than closed (LEP, STS, OSQUAT) chain isotonic strength training exercises. This observation is contradictory to findings from studies comparing open and closed chain isotonic strength training exercises with a similar relative load of 10 to 12 RM in healthy young male subjects (Escamilla et al. 1998; Andersen et al. 2006), which suggested that open chain isotonic exercises generated higher voluntary quadriceps muscle activity levels than closed chain isotonic exercises. Generally, it was reported that while extending the knee joint, subjects generated higher voluntary quadriceps muscle activity near maximal knee joint extension in open compared to closed chain isotonic strength training exercises (Escamilla et al. 1998; Andersen et al. 2006). This discrepancy between the present and previous studies (Escamilla et al. 1998; Andersen et al. 2006) may be explained by 2 factors. First, our sample size was not estimated to determine the difference in voluntary peak quadriceps muscle activity between open and closed chain isotonic strength training exercises. Second, we analyzed voluntary peak quadriceps muscle activity during the strength training exercises, while previous studies investigated the voluntary peak quadriceps muscle activity through the entire (Andersen et al. 2006) or specific range of motion interval (Escamilla et al. 1998).

Isotonic strength training exercises (LEP, STS, OSQUAT, KEM, KEE) elicited higher voluntary peak quadriceps muscle activity compared to the isometric strengthening exercise (SLR). Open chain, isotonic, short-arc knee extension exercise seems to elicit more voluntary quadriceps muscle activity than SLR (Gryzlo et al. 1994). However, the relative load (e.g. 10 RM) was not the same between the strength training exercises (Gryzlo et al. 1994). Additionally, isotonic strength training exercises have been shown to increase muscle strength more than isometric strengthening exercises during a 6-week training period (Rasch and Morehouse 1957). Finally, the SLR exercise involves a bi-articulate muscle, the rectus femoris muscle, which acts over both the knee and hip joint. Consequently, the 10 RM estimation of the SLR may have been based on the fatigue of the rectus femoris muscle and not the VL or VM. If this was the case, VM and VL would potentially produce relative lower voluntary peak muscle activity during the SLR compared to the isotonic strength training exercises.

#### Voluntary peak hamstring muscle activity during closed chain isotonic strength training exercises

The higher voluntary peak hamstring muscle activity of the OSQUAT may be attributed to more unstable conditions compared to LEP and STS (Schwanbeck et al. 2009). Free weight squat increased hamstring muscle activity compared to a squat performed in the more stable Smith machine, which may be explained by a higher demand of knee flexor muscle activity to stabilize the knee and hip joint in a more unstable environment (Schwanbeck et al. 2009).

### Clinical implications

This is the first EMG study to compare commonly used strength training exercises early after TKA. Patients with TKA are challenged by a considerable decrease of knee-extension strength, which is primarily caused by arthrogenic inhibition of the quadriceps muscle (Mizner et al. 2005a; Rice and McNair 2010; Bade and Stevens-Lapsley 2011; Jakobsen et al. 2014b). Our findings suggest that home-based strength training exercises performed with elastic bands or patients’ own body weight is a non-inferior alternative to machine-based strength training exercises when it comes to voluntary peak quadriceps muscle activity. Although superiority of the simpler forms of strength training over machine-based strength training was demonstrated for voluntary peak quadriceps muscle activity in the present study, a conservative interpretation of non-inferiority is in place. That is, differences in the muscle activity/joint angle-relationship and potentially different long-term response between these types of exercises make a conservative interpretation seem the right choice.

The above implies that strength training exercises early after TKA could be performed at home without acquisition of more expensive strength training machines and the need for transport to a rehabilitation center or a similar training facility. Clinicians may instruct patients in simple home training exercise programs (Picorelli et al. 2014) with fewer exercises to enhance adherence (Henry et al. 1999). If this is the case, the KEE combined with STS or OSQUAT would be an appropriate choice. Thereby, the clinicians will take into account the voluntary peak activity of both the quadriceps and hamstring muscles. Additionally, the closed kinetic chain exercises STS and OSQUAT likely facilitate the transfer to important daily activities such as rising from a chair. Finally, home-based strength training exercises may be performed with a potential higher training frequency and promote greater self-management, as patients do not have to attend training at an out-patient rehabilitation facility. This is important as it has been suggested that an increase in the training frequency with fewer sets per muscle group may increase muscle strength over time (Dankel et al. 2017) and, thereby, counteract the pronounced knee-extension muscle strength loss attributed to impaired voluntary quadriceps muscle activity after TKA.

### Limitations

As this study investigated the voluntary peak quadriceps and hamstring muscles activity, we are not able to determine whether home-based would be superior to machine-based strength training exercises in increasing knee-extension strength and functional performance in a long-term rehabilitation intervention in patients with TKA. Likely, there may be no difference between home-based or machine-based (supervised) strength training exercises in increasing knee-extension strength or functional performance as recent studies have suggested that home-based rehabilitation seems to be equally effective as out-patient supervised rehabilitation in patient with TKA (Ko et al. 2013; Han et al. 2015).

Whether other strength training exercises would elicit higher voluntary peak quadriceps muscle activity remains unanswered. The standardization of the hip and ankle joint may impose different results for the bi-acticular hamstring muscles. It is important to emphasize that our study included patients with unilateral primary TKA 4 to 8 weeks postoperatively with pronounced CNS activation failure of the muscles in the lower limb (Mizner et al. 2005a; Rice and McNair 2010). A different result may have arisen, if we included patients before or after this pre-determined time frame 4 to 8 weeks post-TKA, patients with different knee pathologies or healthy subjects. Generally, progressive strength training is recommended to increase muscle strength (Delorme 1945; Kraemer et al. 2002). The progression of the simple home-based strength training exercise (KEE, STS, OSQUAT) may be challenged. This is apparent in STS and OSQUAT, which mostly rely on the patients’ self-perceived weight on the operated leg and unloading of the uninvolved leg corresponding to e.g. 10 RM. The progression in the absolute training load in the machine-based strength training exercise would be managed by choosing more weight stacks on the machine. Consequently, patients need a thorough instruction in how to progress when they perform the simple home-based strength training exercises.

To investigate differences between strength training exercises early after TKA, we used the primary outcome voluntary peak quadriceps muscle activity with a 500 ms RMS window to minimize sudden changes in the muscle activity (Serner et al. 2014). Consequently, voluntary muscle activity through the entire knee joint ROM was not investigated.

Some patients exceeded the 100 %EMGmax, which implies that the maximum voluntary peak quadriceps muscle activity was not reached during the MVIC testing. Consensus of the most appropriate method of normalization of EMG data is lacking (Halaki and Ginn 2012). Using a MVIC performed at a specific knee joint angle position to obtain a EMG reference level to evaluate a dynamic task, such as a strength training exercise, has been debated (Halaki and Ginn 2012). To address this problem, it has been recommended to collect EMG reference levels at every knee joint angle position of interest for each muscle during a dynamic task (Mirka 1991; Halaki and Ginn 2012). This method would be time consuming and fatiguing patients with early TKA (Mirka 1991). We choose to test the MVIC 3 times in 60 degrees of knee joint flexion. This procedure has been suggested to elicit the highest maximum knee extension strength (Haffajee et al. 1972; Halaki and Ginn 2012), can be performed by patients early after TKA (Jakobsen et al. 2011, 2014b), and has been widely used (Andersen et al. 2006; Jakobsen et al. 2012).

## Conclusion

Contrary to the study hypothesis, strength training exercises in more simple forms, using elastic bands or patients’ own bodyweight, elicited higher voluntary peak quadriceps muscle activity than strength training exercises in machines in patients early after TKA. These simple form strength training exercises can be considered for home-based rehabilitation following TKA to target the well-known voluntary quadriceps muscle activation deficit.

## Additional file

Additional file 1: Exercise description. (DOCX 1742 kb)

## Data Availability

The datasets used and analyzed during the current study are available from the corresponding author on reasonable request.

## Abbreviations

%EMGmax: Normalized electromyography
BF: Biceps femoralis
CI: Confidence interval
EMG: Electromyography
KEE: Knee extensions with elastic band
KEM: Knee extensions in machine
LEP: Leg press in machine
OSQUAT: One-legged squat
RM: Repetition maximum
ROM: Range of motion
SD: Standard deviation
SE: Standard error of the mean
SLR: Straight leg raise
ST: Semitendinosus
STS: Sit-to-stand
TKA: Total knee arthroplasty
VL: Vastus lateralis
VM: Vastus medialis
