# Supplementary material for: Quadriceps muscle activity during commonly used strength training exercises shortly after total knee arthroplasty: implications for home-based exercise-selection
Source: J Exp Orthop. 2019 Jul 2;6:29. doi: 10.1186/s40634-019-0193-5 (PMC6606685; doi:10.1186/s40634-019-0193-5)
Supplement: Supplementary file 1 — Exercise description. (DOCX 1742 kb) [file 40634_2019_193_MOESM1_ESM.docx]

| **Additional file 1. Exercise description** | | | | |
| --- | --- | --- | --- | --- |
| Exercise | Starting position | Activity | Load (10 RM) | Exercise equipment |
| Knee extensions in machine (KEM)   | The patients sat in a unilateral knee-extension machine with 90 degrees of flexion in the hip. The roller was placed just above the ankle joint. Patients held the handles on the machine during the exercise. | The patients started the exercise from maximal possible active knee extension to knee flexion. | The load was based on weight stacks on the machine. If needed, smaller weights were placed upon the weight stacks on the machine. The smallest weight increment was 0.5 kilo. | Knee-extension machine with weight stacks (Technogym, Silver Line, Gambettola, Italy).  Ankle weight cuffs. |
| Knee extensions with elastic band (KEE)   | The patients sat upon a high table with 90 degrees of flexion in the hip, and a wedge cushion under the thigh with the highest end of the cushion aligned with the edge of the table. Patients held around the edge of the table. The elastic band was placed just proximal of the ankle with a small pillow/cloth underneath the band to prevent discomfort. | The patients started the exercise from maximal possible active knee extension to knee flexion. | The load was based on elastic bands. The ends of the band was attached to each other, looped and placed under the legs of the table. To find the load, an elastic band with the right resistance was chosen. If needed, 2 elastic bands with different resistance, or the same elastic band doubled, were used. | Elastic bands with resistance of medium (red), heavy (green), extra heavy (blue), and special heavy (black) (TheraBand, Akron, OH,US). |
| Leg press in machine (LEP)   | The patients sat in the leg press machine with the foot placed on the foot support in a standardized position. The seat was adjusted to enable 90 degrees of knee joint flexion. The patients held the handles of the machine during the exercise. | The patients started the exercise from maximal possible active knee extension, maximum 0 degrees, to knee flexion. The uninvolved leg remained relaxed during the exercise. | The load was based on weight stacks on the machine. If needed, a smaller 5-kilo weight was placed upon the weight stacks on the machine. The smallest weight increment was 5 kilos. | Leg press machine with weight stacks and an additional 5-kilo weight.  (Technogym, Element Line, Gambettola, Italy). |
| One-legged squat (OSQUAT)   | The patient placed the operated leg in front of the non-operated leg in a standardized position. The tip of the foot of the non-operated leg was placed on a bathroom scale fitted into a custom-made squared wooden board. | The patients started the exercise from maximal possible active knee extension, maximum 0 degrees, to knee flexion. If needed, the patients were allowed to maintain their balance and to prevent a fall by holding the researcher’s hand with a light touch. | The load was based on the maximum body weight the patient could place on the operated leg, while doing at least a 70 degrees knee joint range of motion. The amount of kilo placed on the non-operated leg was registered on a weight scale fitted into a custom-made wooden platform to indirectly ensure adequate body weight on the operated leg. | None. |
| Sit-to-stand (STS)   | The patient non-operated leg was placed on a bathroom scale fitted into a custom-made squared wooden board. The operated leg was place either in front, parallel or behind the non-operated leg. A normal chair was positioned behind the patient. | The patients started the exercise from maximal possible active knee extension, maximum 0 degrees, to knee flexion. If needed, the patients were allowed to maintain their balance and prevent a fall by holding the researcher’s hand with a light touch. | The load was based on the maximum body weight the patient could place on the operated leg, while doing at least a 70 degrees knee joint range of motion. The amount of kilo placed on the non-operated leg was registered on a weight scale fitted in a designated room in a custom-made wooden platform to indirectly ensure adequate body weight on the operated leg. Furthermore, the toe-to-toe distance between legs was registered. | A normal chair, seat height 45 centimeter. |
| Straight leg raise (SLR)   | The patients lay on the back with the non-operated knee flexed 90 degrees and the foot placed on the couch. The operated knee was maximally extended. | The patients flexed the hip of the operated leg 60 degrees, indicated by a piece of tape on the adjacent wall, with the knee maximally extended and lowered the leg down and up again. The leg was not allowed to rest on the couch during the exercise. | The load was based on ankle cuff weight attached just proximal to the ankle joint. The smallest weight increment was 0.5 kilo. | Ankle weight cuffs. |
